# Exercise-Induced Meat Quality Improvement Is Associated with an lncRNA-miRNA-mRNA Network in Tibetan Sheep

**DOI:** 10.3390/biology15020158

**Published:** 2026-01-16

**Authors:** Pengfei Zhao, Zhiyong Jiang, Xin He, Ting Tian, Fang He, Xiong Ma

**Affiliations:** 1 Gansu Key Laboratory of Biodiversity in Gannan Plateau, Faculty of Chemistry and Life Sciences, Gansu Minzu Normal University, Hezuo 747000, China; 0700090@gnun.edu.cn (P.Z.); 2024010259@stu.gnun.edu.cn (X.H.); 2025010517@stu.gnun.edu.cn (T.T.); 2025010515@stu.gnun.edu.cn (F.H.); 2 Luqu County Animal Disease Prevention and Control Center, Gannan 747000, China; 13893977810@163.com

**Keywords:** biceps femoris, muscle fiber type conversion, intramuscular fat, ceRNA network

## Abstract

Tibetan sheep, a unique breed indigenous to the Qinghai–Tibet Plateau, exhibit remarkable adaptations to high-altitude hypoxia, and their muscle quality is a key economic determinant. However, the molecular mechanisms by which exercise regulates meat quality in this breed remain poorly understood. This study aimed to systematically investigate the effects of different exercise volumes on the biceps femoris muscle of Tibetan sheep, integrating histological analysis with high-throughput transcriptome sequencing. We compared a low-exercise group with a high-exercise group and found that long-term endurance exercise resulted in phenotypic changes suggestive of a shift toward oxidative muscle fiber characteristics. This adaptation was characterized by significantly reduced muscle fiber diameter and cross-sectional area, alongside a crucial increase in intramuscular fat content, collectively enhancing meat tenderness, flavor, and juiciness. Transcriptomic analysis revealed extensive gene expression reprogramming, identifying 208 mRNAs and 490 lncRNAs that were differentially expressed and primarily associated with muscle fiber transition and energy metabolism. Furthermore, we constructed a putative lncRNA-miRNA-mRNA competing endogenous RNA network based on expression correlations and bioinformatic predictions, highlighting potential key regulatory axes such as LOC105603384/miR-16-z/*MYLK3*, LOC121820630/miR-381-y/*NOX4*, and LOC132659150/oar-miR-329a-3p/*NF1*. These findings provide a new perspective on the molecular basis of exercise-induced muscle adaptation in high-altitude animals and offer a solid theoretical framework for improving meat quality through scientific livestock management.

## Simple Summary

This study addresses the unknown problem of how physical exercise influences meat quality in Tibetan sheep, a unique breed living on the Qinghai–Tibet Plateau. Our goal was to compare sheep kept in limited spaces with those that walked long distances on pastures. We found that active sheep developed finer muscle fibers and higher fat content, which makes their meat more tender, juicy, and flavorful. We also identified specific genetic mechanisms that control these beneficial changes. This work has social value as it guides farmers to improve meat quality through better management practices like grazing. This helps provide better food and promotes the sustainable development of the livestock industry.

## 1. Introduction

As a unique livestock species indigenous to the Qinghai–Tibet Plateau, Tibetan sheep have developed distinct genetic adaptations and physiological regulatory mechanisms to high-altitude hypoxia, through long-term natural selection and artificial domestication [1,2,3]. They represent a precious genetic resource for China’s livestock industry. However, with the implementation of policies such as grazing bans, rotational grazing, and grazing suspension, Tibetan sheep breeding has gradually transitioned from a traditional grazing mode to a pen-feeding mode, leading to changes in meat quality. In livestock production, muscle quality is a key determinant of an animal’s economic value, as it directly influences the tenderness, flavor, juiciness, and nutritional value of the meat [4,5]. The basic structural and functional unit of skeletal muscle is the muscle fiber [6], which is primarily categorized into oxidative (Type I) and glycolytic (Type II) based on their metabolic and contractile properties [7]. These types exhibit distinct characteristics: oxidative fibers generally possess smaller diameters, are rich in mitochondria, and exhibit higher intramuscular fat (IMF) deposition, whereas glycolytic fibers are larger in diameter and have lower IMF content [6,7,8,9]. Therefore, the proportional composition of different muscle fiber types within a specific muscle tissue can be effectively inferred from phenotypic indicators such as muscle fiber diameter, cross-sectional area, and IMF content. Furthermore, smaller muscle fiber diameters are associated with reduced shear force and connective tissue density, which enhances tenderness. Concurrently, IMF content determines flavor intensity and water-holding capacity, thereby improving juiciness. Therefore, these metrics can further elucidate the quality characteristics of the meat [10,11].

Exercise is one of the most direct and important environmental factors regulating the structure and function of skeletal muscle [6,12,13,14,15,16]. Grazing and pen-feeding represent two distinct patterns of exercise: grazing sheep require long-duration, long-distance walking for foraging, making their exercise pattern more akin to endurance training; whereas pen-feeding sheep have limited space for activity, resulting in a significantly reduced level of exercise. Numerous studies have shown that exercise of varying intensities can induce adaptive transformations in muscle fiber types [13,14,15,16]. For example, endurance exercise typically promotes the conversion of glycolytic muscle fibers to oxidative fibers [14,15,16,17]. However, there is currently a lack of in-depth research systematically comparing the effects of different exercise volumes on the muscle phenotype, particularly the proportional composition of muscle fibers, in Tibetan sheep, a livestock breed unique to high-altitude regions. Furthermore, the specific mechanisms by which ceRNA regulatory networks mediate exercise-induced muscle fiber adaptation in Tibetan sheep remain largely unexplored.

With the development of high-throughput sequencing technology, transcriptomics has provided a powerful tool for dissecting the molecular regulatory networks of complex life processes [18,19]. In muscle development and metabolic regulation, messenger RNA (mRNA), as the direct template for protein synthesis, has changes in its expression level that directly reflect the functional state of the cell [20,21]. Meanwhile, microRNA (miRNA) and long non-coding RNA (lncRNA), as two important classes of non-coding RNAs, play a crucial role in processes such as myocyte proliferation, differentiation, and muscle fiber type conversion, primarily through post-transcriptional regulation [22,23,24,25]. Among these, the competing endogenous RNA (ceRNA) hypothesis reveals a complex regulatory model: lncRNAs can act as “molecular sponges”, competitively binding to miRNAs, thereby relieving the inhibitory effect of miRNAs on their target mRNAs and forming a lncRNA-miRNA-mRNA regulatory network [26]. Although previous studies largely conducted in other species have confirmed that ceRNA networks play a core regulatory role in myogenesis and muscle fiber type determination [27,28], their specific roles in the process of exercise-induced remodeling of muscle fiber composition in Tibetan sheep remain unexplored.

Accordingly, this study selected Tibetan sheep from a pen-feeding (low exercise, LE) group and a grazing (high exercise, HE) group as the research subjects. The biceps femoris muscle, a major locomotive muscle in the hind limb, was used as the experimental material. This study aims to systematically reveal the effects of exercise volume on the muscle quality of Tibetan sheep and its underlying molecular mechanisms through the following levels: First, by using histological methods, we compared phenotypic differences in the biceps femoris muscle between the two groups, including myofibril diameter, muscle fiber diameter, cross-sectional area, and IMF content. This will allow us to infer the impact of exercise volume on the proportional composition of muscle fiber types and subsequently evaluate differences in meat quality. Second, utilizing transcriptome sequencing technology, we will screen for differentially expressed lncRNAs (DElncRNAs), miRNAs (DEmiRNAs), and mRNAs (DEmRNAs) between the two groups. Finally, based on the DERNAs and bioinformatics predictions, we constructed an lncRNA-miRNA-mRNA ceRNA regulatory network related to muscle fiber type conversion. By integrating morphological and transcriptomic evidence, this research seeks to systematically elucidate the molecular mechanisms by which exercise influences the muscle fiber composition in Tibetan sheep. This work will not only provide a new perspective for a deeper understanding of environmental adaptation in high-altitude animals but also offer a crucial theoretical basis and data support for improving the muscle quality of Tibetan sheep through scientific feeding and management strategies, thereby promoting the sustainable development of the plateau livestock industry.

## 2. Materials and Methods

### 2.1. Experimental Design and Sample Collection

Three-year-old castrated Tibetan rams, sourced from Xiahe County, Gannan Tibetan Autonomous Prefecture, Gansu Province, China, were allocated into an HE group and an LE group. The HE group rams traversed a daily 20 km round-trip between their pen and pasture, a regimen maintained for six months. Conversely, the LE group was housed in semi-open pens, with five sheep per pen, maintained under a natural light cycle with free access to water. The five sheep in the HE group returned to similar pens after grazing. To ensure experimental validity, both groups were provided with an identical diet, with the LE group receiving forage from the same pasture. The primary intended variable was the daily, long-distance activity performed by the HE group compared to the restricted condition of the LE group. Each group consisted of five biological replicates. Although modest, this sample size was determined based on similar high-throughput transcriptomic studies in livestock muscle research, where n = 5 has been shown to provide adequate statistical power for identifying significant differential expression [29,30,31].

All animals were processed according to standard industry protocols, involving electrical stunning followed by rapid exsanguination to ensure a swift death. Subsequently, the biceps femoris muscle was promptly and precisely excised from the left side of each carcass. Samples were then divided: a portion was fixed in 3% glutaraldehyde for myofibril diameter measurement, another in 4% paraformaldehyde for muscle fiber diameter and cross-sectional area analysis, and the remainder was snap-frozen in liquid nitrogen for IMF content determination and transcriptome sequencing.

### 2.2. Morphological Structure and Intramuscular Fat Content Analysis

For this study, five samples from each of the LE and HE groups were selected to analyze the biceps femoris myofibril diameter via transmission electron microscopy. Samples preserved in 3% glutaraldehyde at 4 °C underwent post-fixation with 1% osmium tetroxide. This was followed by dehydration through a graded ethanol series (30%, 50%, 70%, 80%, 90%, 95%, and 100%). Subsequently, the samples were infiltrated with mixtures of ethanol and epoxy resin at volume ratios of 3:1, 1:1, and 1:3 for 40 min per step. The samples were then embedded in epoxy resin and sectioned into ultrathin slices using an EM-UC7 ultramicrotome (Leica, Wetzlar, Germany). These slices were stained with uranyl acetate and lead citrate for 15 to 20 min. Finally, the ultrathin sections stained with uranyl acetate and lead citrate were imaged using a JEM-1400FLASH TEM (JEOL, Tokyo, Japan). For each sample, the myofibril diameter of 20 myofibrils was manually and randomly measured from fields of view at 12,000× magnification using Image-Pro Plus 6.0 software.

The biceps femoris muscle fiber diameter and cross-sectional area were assessed using conventional Hematoxylin & Eosin staining. Samples fixed in 4% paraformaldehyde were rinsed under running water and dehydrated through a graded series of ethanol solutions (75%, 85%, 95%, and 100%). After dehydration, the tissues were cleared in xylene and embedded in paraffin to form blocks. These blocks were sectioned at a thickness of 5 μm using a Leica 2016 rotary microtome (Leica, Wetzlar, Germany). The resulting sections were deparaffinized in xylene, rehydrated through a graded ethanol series, and stained with hematoxylin for 5 min followed by eosin for 1 min according to standard protocols. The stained sections were examined and imaged at 400× magnification with a BA210 digital microscope (Motic, Xiamen, China). Using Image-Pro Plus 6.0 software, the diameter and cross-sectional area of 10 muscle fibers were randomly measured for each sample. To ensure representativeness, the diameter and cross-sectional area of 10 muscle fibers were randomly selected from multiple, non-overlapping fields of view for each sample. Although the fiber count is modest, this random sampling strategy effectively captured tissue heterogeneity and yielded statistically robust results.

The IMF content of the biceps femoris was quantified via the Soxhlet extraction method, employing petroleum ether as the solvent. Muscle samples stored in liquid nitrogen were freeze-dried for 48 h in a freeze dryer (Labconco, Kansas, KS, USA) until fully dehydrated. The freeze-dried samples were then ground into a homogeneous fine powder with a tissue grinder (Bertin Technologies, Montigny-le-Bretonneux, France). Approximately 5 g of the muscle powder was accurately weighed, wrapped in filter paper, and placed in a Soxhlet extractor (Thermo Fisher Scientific, Waltham, MA, USA). Fat was extracted using petroleum ether for 8 h to ensure complete removal. The samples were then dried and re-weighed to calculate the IMF content as the percentage of fat weight relative to the initial sample weight, with each measurement performed in triplicate [32].

Statistical analysis was performed using biological replicates. Specifically, for morphological measurements, the mean value for each individual animal was calculated prior to analysis. We assessed the normality of data distribution using the Shapiro–Wilk test and homogeneity of variances using Levene’s test. Since the data satisfied the assumptions of normality and homogeneity, we utilized a two-tailed independent samples Student’s *t*-test. SPSS 25.0 (SPSS, Chicago, IL, USA) was utilized for *t*-test analysis to compare these group means of myofibril diameter, muscle fiber diameter and cross-sectional area, and IMF content. Results are presented as mean ± standard deviation, and a *p* < 0.05 was considered to indicate statistical significance.

### 2.3. Transcriptome Sequencing and Transcript Assembly

Total RNA was extracted from the biceps femoris tissue of five Tibetan sheep from each of the LE and HE groups using a TRIzol kit (Invitrogen, Carlsbad, CA, USA). RNA integrity and purity were assessed using an Agilent 2100 Bioanalyzer (Agilent Technologies, Palo Alto, CA, USA). Only samples with an RNA Integrity Number ≥ 7.0 and a 28S/18S ratio ranging from 1.8 to 2.0 were included in subsequent library construction to ensure high-quality sequencing data. Ribosomal RNA (rRNA) was subsequently removed from the RNA samples using a Ribo-Zero Gold rRNA kit (Illumina, San Diego, CA, USA). The remaining mRNA was then enriched with SPRI purification beads (Beckman Coulter, Brea, CA, USA) and fragmented via high-temperature treatment. Using the fragmented mRNA as a template, cDNA libraries were constructed with the NEBNext Ultra RNA Library Prep Kit (New England Biolabs, Ipswich, MA, USA). The purified double-stranded cDNA underwent sequential end-repair, A-tailing, and adapter ligation, followed by PCR amplification and purification with AHTS DNA Clean Beads (Vazyme, Nanjing, China). Finally, the prepared libraries were sequenced on an Illumina NovaSeq X Plus platform (Illumina, San Diego, CA, USA) using a paired-end 150 strategy.

To ensure data quality, the raw reads were processed using FASTQ v.0.24.0 for quality control [33]. This step involved the removal of reads containing adapters, reads with a N content exceeding 10%, reads composed entirely of A bases, and reads where over 50% of the bases had a quality score of Q ≤ 20, resulting in a set of clean reads. Subsequently, these clean reads were aligned to an rRNA database using Bowtie2 v.2.5.4 [34], and any mapped reads were discarded. The remaining unmapped reads were then aligned to the sheep reference genome (ARS-UI_Ramb_v3.0) with HISAT2 v.2.2.1 [35]. Finally, transcript assembly was performed using Stringtie v.1.3.4 [36,37].

### 2.4. Differential Expression and Functional Enrichment Analysis

To account for the significant variability among lncRNA isoforms produced by the same gene, the analysis was performed at the transcript level. First, known lncRNAs were downloaded from the NONCODE, Ensembl, and NCBI databases. The transcripts assembled by Stringtie v.1.3.4 were then filtered to retain sequences with a length of ≥200 bp and two or more exons. The coding potential of these novel transcripts was subsequently predicted using CPC v.2 [38], CNCI v.0.9-r2 [39], and FEELnc v.0.2.1 [40]. The intersection of transcripts identified as non-coding by all three tools was taken as the final prediction set. Transcript abundance was quantified using transcripts per million reads (TPM) values calculated with the RSEM v.1.3.3 software [41]. Subsequently, differential expression analysis of mRNAs and lncRNAs between the LE and HE groups was conducted using the DESeq2 v.1.34.0 using the expected raw counts generated by RSEM v.1.3.3 as input [42]. To stabilize log2 fold change (FC) estimates for genes with low counts, the lfcShrink function (apeglm) was applied. DEmRNAs were defined by a false discovery rate below 0.05 and an absolute FC exceeding 2. For DElncRNAs, the thresholds for significance were a *p* less than 0.05 and an absolute FC greater than 1.5. These criteria were selected based on the distinct expression characteristics of lncRNAs. Since lncRNAs generally exhibit lower abundance and function as fine-tuning regulators, the FC > 1.5 threshold was adopted to increase sensitivity and capture potentially key regulators that might be missed using the stricter FC > 2 criterion [43,44].

Functional enrichment analysis, including Gene Ontology (GO) term and Kyoto Encyclopedia of Genes and Genomes (KEGG) pathway analyses, was conducted on the DEmRNAs using GOATOOLS v.1.3.1 [45,46] and KOBAS v.2.0 [47,48]. Significantly enriched GO terms and KEGG pathways were determined by Fisher’s exact test, with a *p* of <0.05 set as the threshold for statistical significance [49].

### 2.5. Constructing the ceRNA Network

To identify the specific lncRNA-miRNA-mRNA axes responsible for the meat quality improvements observed in this study, we integrated expression data of DElncRNAs, DEmRNAs, and existing DEmiRNAs. First, potential lncRNA-miRNA and miRNA-mRNA regulatory pairs were screened based on a significant negative correlation (reflecting a regulatory targeting relationship) in their expression levels, using a Spearman rank correlation coefficient (SCC) < −0.5 and a *p* < 0.05 as the threshold. Meanwhile, lncRNA-mRNA pairs with a Pearson correlation coefficient (PCC) > 0.85 were identified as positively co-regulated, indicating a synergistic relationship typical of the ceRNA mechanism. The final lncRNA-miRNA-mRNA ceRNA network was constructed by integrating these criteria, connecting an lncRNA and an mRNA if they shared a negatively correlated miRNA and were themselves positively correlated [26,50]. This network facilitates the identification of key regulatory nodes that drive the phenotypic meat quality changes, such as increased tenderness and IMF content, in the biceps femoris of Tibetan sheep.

## 3. Results

### 3.1. Phenotypic Differences in the Biceps Femoris

**Figure 1:**
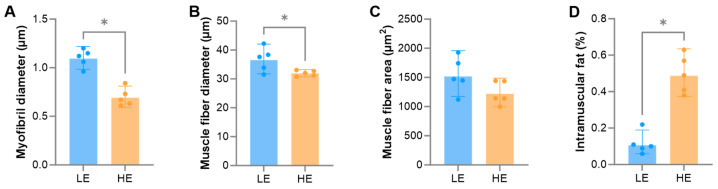
Morphological and biochemical characteristics of the biceps femoris muscle in Tibetan sheep under different exercise regimes: (**A**) Myofibril diameter, (**B**) muscle fiber diameter, (**C**) muscle fiber cross-sectional area, (**D**) intramuscular fat (IMF) content. Analysis was based on five biological replicates (n = 5 rams per group). To avoid pseudoreplication, individual animal means were calculated from 20 randomly selected myofibrils (**A**), 10 randomly selected muscle fibers (**B**,**C**), or three technical replicates for IMF (**D**) prior to statistical analysis. Statistical significance between the low exercise (LE) and high exercise (HE) groups was determined using a *t*-test, * indicates *p* < 0.05.

The effects of exercise on the myofibril diameter, muscle fiber diameter, muscle fiber cross-sectional area, and IMF content of the biceps femoris in Tibetan sheep are shown in Figure 1. The myofibril diameter and muscle fiber diameter were significantly lower in the HE group than in the LE group (*p* < 0.05), which is biologically associated with improved meat tenderness. Although the muscle fiber cross-sectional area showed only a tendency to be lower than in the LE group (*p* = 0.094), this trend supports the shift towards finer muscle fibers and suggests a potential improvement in texture. In contrast, the IMF content was significantly higher (*p* < 0.05), a crucial determinant of meat flavor and juiciness. Collectively, these morphological and biochemical changes suggest a shift in muscle phenotype characteristics toward those associated with oxidative fibers.

### 3.2. Summary of Sequencing Data

**Figure 2:**
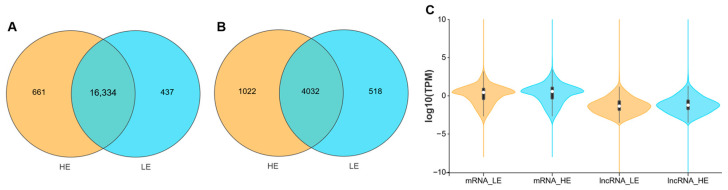
Overview of expressed transcripts in the biceps femoris of Tibetan sheep. Venn diagram showing the overlap of expressed mRNAs (**A**) and lncRNAs (**B**) between the LE and HE groups, (**C**) Violin plot comparison of the overall expression levels (log10 TPM) between mRNAs and lncRNAs. Transcripts with TPM > 0.01 were considered expressed.

A total of ten cDNA libraries were generated from the biceps femoris of Tibetan sheep. These libraries underwent RNA sequencing, and a summary of the resulting data is provided in Table 1. On average, the LE and HE groups yielded 81,866,550 and 89,725,502 raw reads, respectively. After a filtering process to remove reads containing adapters, reads with a N content exceeding 10%, reads composed entirely of A bases, and reads with over 50% of their bases having a quality score of Q ≤ 20, the LE and HE groups retained an average of 81,478,946 and 89,316,973 clean reads, respectively. Subsequently, after discarding reads that mapped to rRNA, the remaining clean reads for the LE and HE groups were 79,869,272 and 88,336,808. Of these remaining clean sequences, an average of 70,187,050 (87.88%) from the LE group and 79,813,459 (90.35%) from the HE group were successfully aligned to the reference genome (ARS-UI_Ramb_v3.0). The unique mapping rates for the LE and HE groups were 77.05% and 83.39%, respectively. Expressed mRNAs were identified using a threshold of TPM > 0.01. This analysis detected 16,771 and 16,995 expressed mRNAs in the biceps femoris of the LE and HE groups, respectively. Among these, 16,334 mRNAs were commonly expressed in both groups (Figure 2A). Similarly, applying a TPM threshold of >0.01 to define expressed lncRNAs led to the identification of 4550 and 5054 such transcripts in the LE and HE groups, respectively. Of these, 4032 lncRNAs were co-expressed in both groups (Figure 2B). Moreover, lncRNAs exhibited lower expression levels compared to mRNAs (Figure 2C).

### 3.3. Differential Expression of mRNAs and lncRNAs

**Figure 3:**
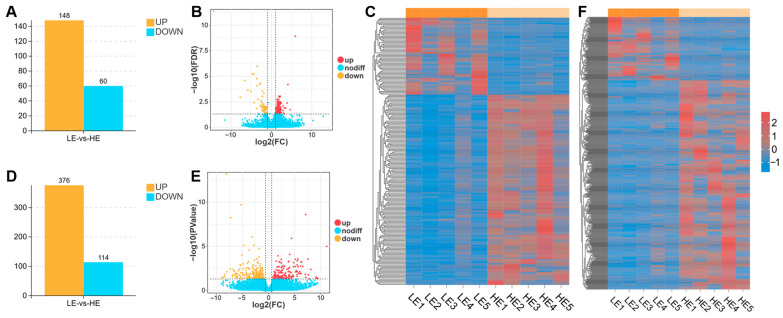
Differential expression profiles of mRNAs and lncRNAs in the biceps femoris of Tibetan sheep between the LE and HE groups. The DEmRNAs (**A**–**C**) and DElncRNAs (**D**–**F**) between these two groups are visualized through bar graphs, volcano plots, and heatmaps. DEmRNAs were identified with a threshold of |fold change| > 2 and FDR < 0.05. DElncRNAs were identified with a threshold of |fold change| > 1.5 and *p* < 0.05.

We compared the expression levels of mRNAs and lncRNAs in the biceps femoris of Tibetan sheep subjected to different exercise volumes. The LE and HE groups comparison pinpointed 208 DEmRNAs, of which 148 were significantly upregulated, and 60 were downregulated (Figure 3A–C). Additionally, this analysis uncovered 490 DElncRNAs, with 376 showing significant upregulation and 114 showing downregulation (Figure 3D–F).

### 3.4. GO and KEGG Analysis of DEmRNAs

**Figure 4:**
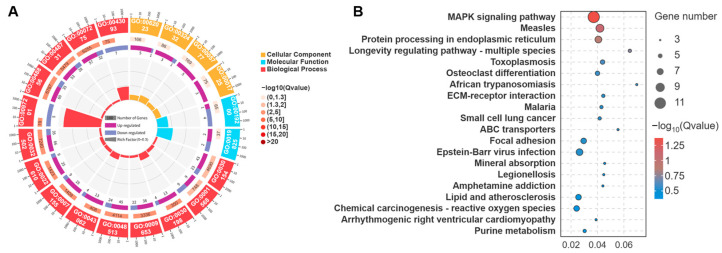
GO and KEGG enrichment analysis of DEmRNAs in the biceps femoris of Tibetan sheep between the LE and HE groups. Key GO terms (**A**) and KEGG pathways (**B**) significantly enriched are shown. Enrichment was determined using Fisher’s exact test with a significance threshold of *p* < 0.05.

GO term annotation revealed that the 208 DEmRNAs between the LE and HE groups were significantly enriched in 58 cellular components, 204 molecular functions, and 973 biological process terms. Notably, key terms related to muscle fiber type conversion and energy metabolism were identified, including collagen-containing extracellular matrix (GO:0062023), actin filament bundle (GO:0032432), peroxisome (GO:0005777), stress fiber (GO:0001725), carbohydrate kinase activity (GO:0019200), oxygen binding (GO:0019825), cell differentiation (GO:0030154), and blood vessel development (GO:0001568) (Figure 4A).

KEGG pathway enrichment analysis further showed these DEmRNAs were significantly enriched in 21 pathways, predominantly involved in muscle fiber type conversion and energy metabolism, such as the MAPK signaling pathway (ko04010), ECM–receptor interaction (ko04512), focal adhesion (ko04510), mineral absorption (ko04978), and chemical carcinogenesis-reactive oxygen species (ko05208) (Figure 4B).

### 3.5. lncRNA-miRNA-mRNA Network Construction

**Figure 5:**
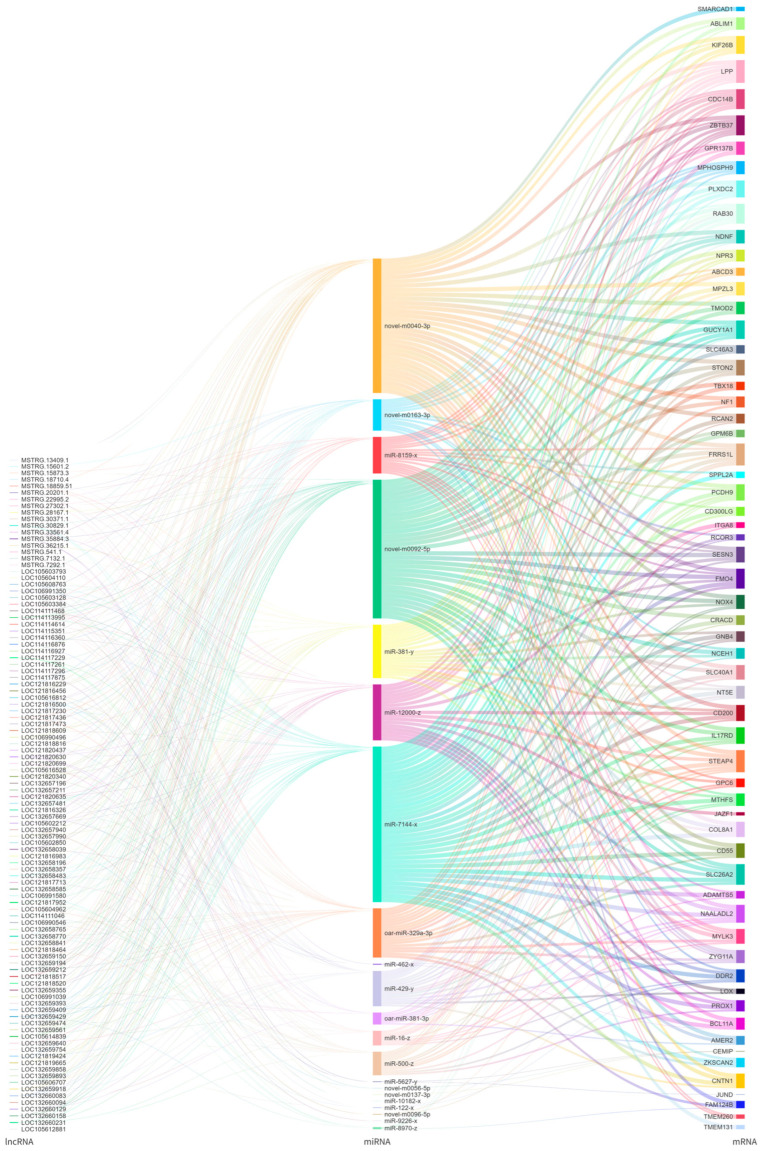
The predicted lncRNA-miRNA-mRNA ceRNA network in the biceps femoris of Tibetan sheep. The Sankey diagram illustrates the putative regulatory interactions involving lncRNAs (left column), miRNAs (middle column), and mRNAs (right column). Connections represent predicted targeting relationships based on expression correlation screening criteria: miRNA-mRNA and lncRNA-miRNA pairs were selected using the Spearman correlation coefficient < −0.5 and *p* < 0.05, while mRNA-lncRNA pairs were selected using the Pearson correlation coefficient > 0.85.

Target prediction for the 35 DEmiRNAs between the LE and HE groups identified 171 DEmRNAs, generating 412 negatively correlated (SCC < −0.5 and *p* < 0.05) miRNA-mRNA pairs. Similarly, 477 DElncRNAs were predicted as targets, forming 1505 lncRNA-miRNA pairs with SCC less than −0.5 and *p* < 0.05. By integrating these interactions, a total of 822 potential ceRNA pairs, characterized by a PCC > 0.85 were ultimately identified. The resulting predictive ceRNA network is visualized in a Sankey diagram (Figure 5) to illustrate the hypothesized regulatory landscape.

## 4. Discussion

The results of this study reveal a significant impact of different exercise volumes on the phenotype of the biceps femoris muscle in Tibetan sheep. Compared to the LE group, Tibetan sheep in the HE group exhibited lower myofibril diameter, muscle fiber diameters and cross-sectional area (Figure 1A–C). Based on the characteristics of muscle fibers, Type I fibers have a smaller diameter, whereas Type II fibers are larger [51,52]. Thus, the observed reduction in muscle fiber diameter in the HE group suggests that long-term endurance exercise may facilitate a transition of muscle fibers towards oxidative characteristics. This transition represents a classic adaptive remodeling in response to sustained energy demand, aimed at enhancing the muscle’s aerobic metabolic capacity and endurance [14,15,16,17]. From a meat quality perspective, a smaller muscle fiber diameter is typically associated with improved tenderness, as finer fibers reduce connective tissue density, resulting in more tender meat [10,11]. The measurement of myofibril diameter was included as a structural indicator of contractile function. Exercise-induced remodeling often involves alterations in myofibril packing density and structure, which are associated with changes in muscle tenderness and texture. More importantly, we found that the IMF content was significantly higher in the HE group than in the LE group (Figure 1D). IMF is a crucial determinant of meat flavor and juiciness [10]. This phenotypic change is strongly associated with the conversion of muscle fiber types. As stated in the Introduction, oxidative muscle fibers possess a greater capacity for IMF deposition [9]. Consequently, exercise synergistically enhances the tenderness, flavor, and juiciness of Tibetan sheep meat by remodeling both the morphological characteristics (reduced diameter) and biochemical composition (increased IMF) of the muscle fibers. This provides direct phenotypic evidence for improving the quality of plateau livestock products through scientific feeding and management strategies, such as moderate grazing.

To further elucidate the molecular mechanisms underlying the aforementioned phenotypic remodeling, we conducted high-throughput transcriptome sequencing analysis. The results revealed that exercise induced extensive gene expression reprogramming in the skeletal muscle of Tibetan sheep, with a total of 208 DEmRNAs and 490 DElncRNAs identified (Figure 3). Numerous studies have demonstrated that mRNA and lncRNA play pivotal roles in skeletal muscle fiber transformation and energy metabolism regulation [24,53,54,55]. In this study, GO and KEGG analyses revealed that the DEmRNAs were primarily associated with muscle fiber transition and energy metabolism (Figure 4). For example, the *MYLK3* gene, annotated to the cell differentiation term and the focal adhesion signaling pathway, can phosphorylate myosin light chains, thereby enhancing muscle contractility [56,57]. The *NOX4* gene, annotated to the actin filament bundle, stress fiber, and oxygen binding terms, as well as the MAPK and chemical carcinogenesis-reactive oxygen species signaling pathways, can catalyze the production of ROS. ROS, in turn, can activate multiple signaling pathways, such as MAPK, thereby influencing the proliferation and differentiation of muscle fibers [58]. Furthermore, this gene promotes the oxidation of glucose and fatty acids, thus regulating muscular performance [59,60]. Meanwhile, the *NF1* gene, annotated to the cell differentiation and blood vessel development terms, promotes skeletal muscle formation and maintains mitochondrial respiratory function [61,62].

lncRNAs can act as molecular sponges, competitively binding with miRNAs to alleviate the inhibitory effect of miRNAs on their target mRNAs [25,26]. This study constructed a predictive lncRNA-miRNA-mRNA interaction network (Figure 5). The results suggest that LOC105603384 may interact with miR-16-z, miR-8159-x, novel-m0040-3p, and oar-miR-329a-3p to potentially alleviate the inhibition on *MYLK3*. LOC121820630 and LOC132657196 may bind with miR-381-y, miR-500-z, oar-miR-381-3p, and novel-m0040-3p to weaken the inhibition on *NOX4*. Meanwhile, LOC132659150 might bind with novel-m0040-3p, novel-m0092-5p, and oar-miR-329a-3p to reduce the inhibition on *NF1*. However, it is important to emphasize that these ceRNA interactions are predicted bioinformatics correlations and require further functional validation to confirm their specific regulatory mechanisms. These lncRNAs and mRNAs were significantly upregulated in the HE group, potentially promoting an increased proportion of oxidative muscle fibers in the biceps femoris of Tibetan sheep to maintain the energy supply for long-distance pastoralism. This is consistent with the observations in this study that the HE group exhibited decreased myofibril diameter, muscle fiber diameter and cross-sectional area, while the IMF content was increased.

Nevertheless, this study has several notable limitations that warrant careful consideration. The relatively small sample size (n = 5 per group) and the exclusive examination of the biceps femoris muscle inevitably constrain the statistical power and limit the generalizability of our findings. While this sample size is consistent with similar exploratory transcriptomic studies, we acknowledge that larger cohorts would be required to validate these results for broader application. Furthermore, the lack of experimental validation is a critical limitation. The proposed ceRNA network is based on bioinformatic predictions and expression correlations, and thus, the specific regulatory mechanisms proposed herein require rigorous functional validation through wet-lab experiments. In addition, the complex nature of grazing introduces potential confounding variables beyond physical activity. While we controlled the diet source by providing the LE group with forage from the same pasture, the actual feed intake of the HE group during grazing was not strictly monitored compared to the provisioned ration for the LE group. Environmental factors, such as UV radiation exposure, temperature, and differences in microclimate, may also influence the phenotype and cannot be entirely disentangled from the effects of exercise. Besides this, histological determination of muscle fiber types in this study relied on H&E staining and diameter measurements, which are proxies for oxidative capacity. We were unable to distinguish specific myosin heavy chain isoforms or fiber subtypes (e.g., Type I vs. II) without immunohistochemical staining, potentially limiting the precision of our fiber type classification. Future research should prioritize validating these key regulatory axes and extending the analysis to other muscle types to confirm these observations.

## 5. Conclusions

In conclusion, this study demonstrates that exercise volume significantly impacts Tibetan sheep muscle quality. Specifically, exercise is associated with a shift from glycolytic to oxidative muscle fibers. This adaptation correlates with reduced fiber diameter and increases IMF content, thereby contributing to enhancing meat tenderness, flavor, and juiciness. At the molecular level, this phenotypic shift is associated with extensive gene expression reprogramming and a predicted lncRNA-miRNA-mRNA ceRNA network, involving key genes such as *MYLK3*, *NOX4*, and *NF1*. Although these findings are currently limited by sample size and biceps femoris specificity, they indicate that optimizing exercise regimens may be an effective strategy to improve meat quality in livestock production.

## Figures and Tables

**Table 1:** Summary of sequencing data.

Altitude	Average Raw Reads	Average Clean Reads	Average Remaining Clean Reads	Average Mapped Reads (%)	Unique Mapped (%)
LE	81,866,550	81,478,946	79,869,272	70,187,050 (87.88%)	61,536,710 (77.05%)
HE	89,725,502	89,316,973	88,336,808	79,813,459 (90.35%)	73,662,081 (83.39%)

## Data Availability

The raw sequencing data used in this study were deposited in the NCBI Sequence Read Archive under BioProject accession numbers PRJNA1289012 and PRJNA1284595.

## Abbreviations

The following abbreviations are used in this manuscript:

IMF	Intramuscular fat
mRNA	Messenger RNA
miRNA	MicroRNA
lncRNA	Long non-coding RNA
ceRNA	Competing endogenous RNA
LE	Low exercise
HE	High exercise
DE	Differentially expressed
rRNA	Ribosomal RNA
TPM	Transcripts per million reads
FC	Fold change
GO	Gene ontology
KEGG	Kyoto encyclopedia of genes and genomes
SCC	Spearman’s rank correlation coefficient
PCC	Pearson correlation coefficient
